# Modeling vegetation community responses to sea-level rise on Barrier Island systems: A case study on the Cape Canaveral Barrier Island complex, Florida, USA

**DOI:** 10.1371/journal.pone.0182605

**Published:** 2017-08-10

**Authors:** Tammy E. Foster, Eric D. Stolen, Carlton R. Hall, Ronald Schaub, Brean W. Duncan, Danny K. Hunt, John H. Drese

**Affiliations:** Ecological Program, Integrated Mission Support Services, Kennedy Space Center, Florida, United States of America; Universidade de Aveiro, PORTUGAL

## Abstract

Society needs information about how vegetation communities in coastal regions will be impacted by hydrologic changes associated with climate change, particularly sea level rise. Due to anthropogenic influences which have significantly decreased natural coastal vegetation communities, it is important for us to understand how remaining natural communities will respond to sea level rise. The Cape Canaveral Barrier Island complex (CCBIC) on the east central coast of Florida is within one of the most biologically diverse estuarine systems in North America and has the largest number of threatened and endangered species on federal property in the contiguous United States. The high level of biodiversity is susceptible to sea level rise. Our objective was to model how vegetation communities along a gradient ranging from hydric to upland xeric on CCBIC will respond to three sea level rise scenarios (0.2 m, 0.4 m, and 1.2 m). We used a probabilistic model of the current relationship between elevation and vegetation community to determine the impact sea level rise would have on these communities. Our model correctly predicted the current proportions of vegetation communities on CCBIC based on elevation. Under all sea level rise scenarios the model predicted decreases in mesic and xeric communities, with the greatest losses occurring in the most xeric communities. Increases in total area of salt marsh were predicted with a 0.2 and 0.4 m rise in sea level. With a 1.2 m rise in sea level approximately half of CCBIC’s land area was predicted to transition to open water. On the remaining land, the proportions of most of the vegetation communities were predicted to remain similar to that of current proportions, but there was a decrease in proportion of the most xeric community (oak scrub) and an increase in the most hydric community (salt marsh). Our approach provides a first approximation of the impacts of sea level rise on terrestrial vegetation communities, including important xeric upland communities, as a foundation for management decisions and future modeling.

## Introduction

Barrier islands are highly susceptible to a rapidly changing climate. Coastal zones are dynamic environments responding across a variety of space and time scales to global, regional and local geomorphologic, oceanographic, and climatic factors [1]. Complicating these influences, human activities during the last 150 years have greatly impacted coastal ecosystems, with approximately 23% of the world’s population living below 100 m elevation and within 100 km of the coast [2]. This demand for space has led to extensive conversion of coastal areas to urban, agricultural, and industrial land cover types putting pressure on remaining natural areas to support valuable ecosystem services such as storm protection, fisheries production, wildlife habitat, recreational use, tourism, and global biodiversity. The distribution and abundance of vegetation communities in the remaining coastal natural areas will be impacted by hydrological alterations associated with climate change including the intensity, duration, and seasonal patterns of rainfall, sea-level rise (SLR), tidal range, and storm surge [3, 4].

**Fig 1:**
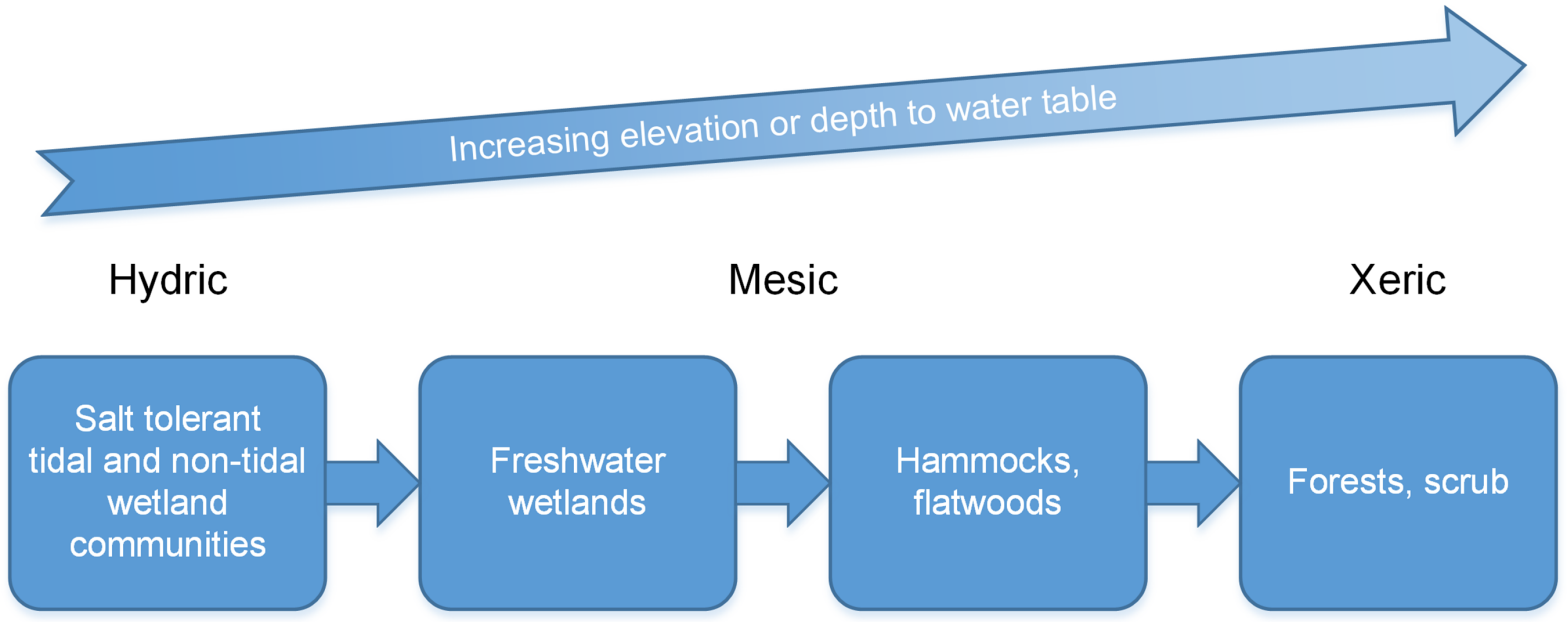
Generalized vegetation community distribution along the elevation gradient in barrier island systems.

Barrier island vegetation communities are primarily organized along the depth to water table gradient that extends from the sea water edge toward higher elevations with salinity influencing communities due to changes in water levels and salt water intrusion [5, 6]. This is expressed as a pattern of zonation which is a function of a species within a guild or community adapting to factors regulated by the frequency and duration of inundation [7]. Zonation is a physiological adaptation to salinity, duration of root inundation, and depth to water table (vadose zone thickness, the non-permanently saturated upper zone of the surficial aquifer), key factors controlling the distribution of vegetation communities across coastal landscapes (Fig 1). The influence of SLR on depth to water table is a function of groundwater elevation relative to the land surface elevation and distance from the shoreline [3, 7, 8]. On barrier islands SLR will directly influence the surficial aquifer elevation and related eco-hydraulic processes such as infiltration, retention, runoff, biogeochemistry, and biodiversity [1, 8–10].

The potential impacts of rapid climate change and SLR on coastal communities and their vital ecosystem services must be addressed because the impacts of anthropogenic pressures have reduced and degraded many of these communities [1]. The importance of the inland displacement of coastal vegetation communities in response to rising water tables and saltwater intrusion associated with climate change induced SLR has been the focus of research for several decades [11–14]. Many coastal communities appear to be resilient to short term extreme events such as hurricanes, floods and storm surges and historically rebound from these disturbances [15]. The long term resilience of coastal communities and their ability to respond to rapid changes in sea level is largely unknown. One approach to examining the impact of SLR on coastal communities is to model community shifts at the local scale.

Many coastal impact models have been created to examine how SLR will affect coastal habitat communities. A review of several types of SLR models classified the applicability of models for purposes varying from international and government policy making to conservation decision making [16]. Sea-level impact models range in terms of spatial resolution, complexity of input and output parameters, and costs. Inundation models are inexpensive, quick and require readily available data sets; these models can provide valuable information for conservation planning, even though they tend to overestimate coastal impacts since feedbacks such as accretion are not included [16]. Other models require more complex input parameters, including geomorphic and biophysical feedbacks or empirical functions that represent these feedbacks, providing a more detailed assessment of the impact of SLR on coastal communities; accounting for the rate at which marshes gain (build) elevation [16–18].

A common characteristic of coastal impact models is that the focus is on the impact of SLR of low-lying vegetation communities; from hydric habitats such as tidal marshes (fresh- and salt- water) to mesic communities. The Sea Level Affecting Marshes Model (SLAMM; EPA, http://www.warrenpinnacle.com) and the Marsh Accretion and Inundation Model (MAIM) [17] are examples of coastal impact models that identify impacts not only on hydric and mesic communities but also on undeveloped dry uplands. The upland xeric habitats of barrier islands play an important role in providing habitat and for species diversity; these upland communities also experience strong anthropogenic land use change pressure [19] Understanding the SLR impact on barrier island vegetation communities ranging from marshes to the upland vegetation communities may provide important information for land management decision making. As sea level rises, the lower lying vegetation communities may be able to migrate inland, moving up the elevation gradient, while the upland vegetation is limited by the upper elevation of the barrier island. These important upland communities may constrict from their lower elevation bounds as the hydric and mesic vegetation communities shift up the elevation gradient.

Our objective was to model how the proportion of vegetation communities (hydric to upland xeric) will shift under three SLR scenarios (0.2 m, 0.4m, and 1.2 m) on the Cape Canaveral Barrier Island Complex (CCBIC). Vegetation response to SLR involves many non-linear complex processes operating simultaneously that require extensive data sets on geomorphic and biophysical feedbacks. For this reason, we utilized a probabilistic approach to develop a first approximation or forecast of potential changes in the vegetation community structure that is driven primarily by relative water level elevation changes with rising sea-level. As the local sea level rises, specific locations on the landscape will experience a decrease of relative elevation with regard to mean water level.

We reasoned that the distribution of existing vegetation communities integrates information about the suite of ecological conditions to which they are adapted. Our rationale for using community elevations to predict future community occurrence following SLR is that there is a direct mechanistic relationship between elevation and community distribution; communities in Florida largely occur along a relative elevation gradient which serves as a proxy for distance to the water table and salt concentration [20–23]. While we acknowledged that this relationship does not fully explain the current distributions on CCBIC, we made the simplifying assumption that vegetation communities are distributed along an elevation gradient that is indicative of the depth to water table [23, 24]. This relationship can be exploited by using the existing distribution of elevations within communities to predict their future distributions caused by relative elevation changes due to SLR.

## Methods

### Study site

**Fig 2:**
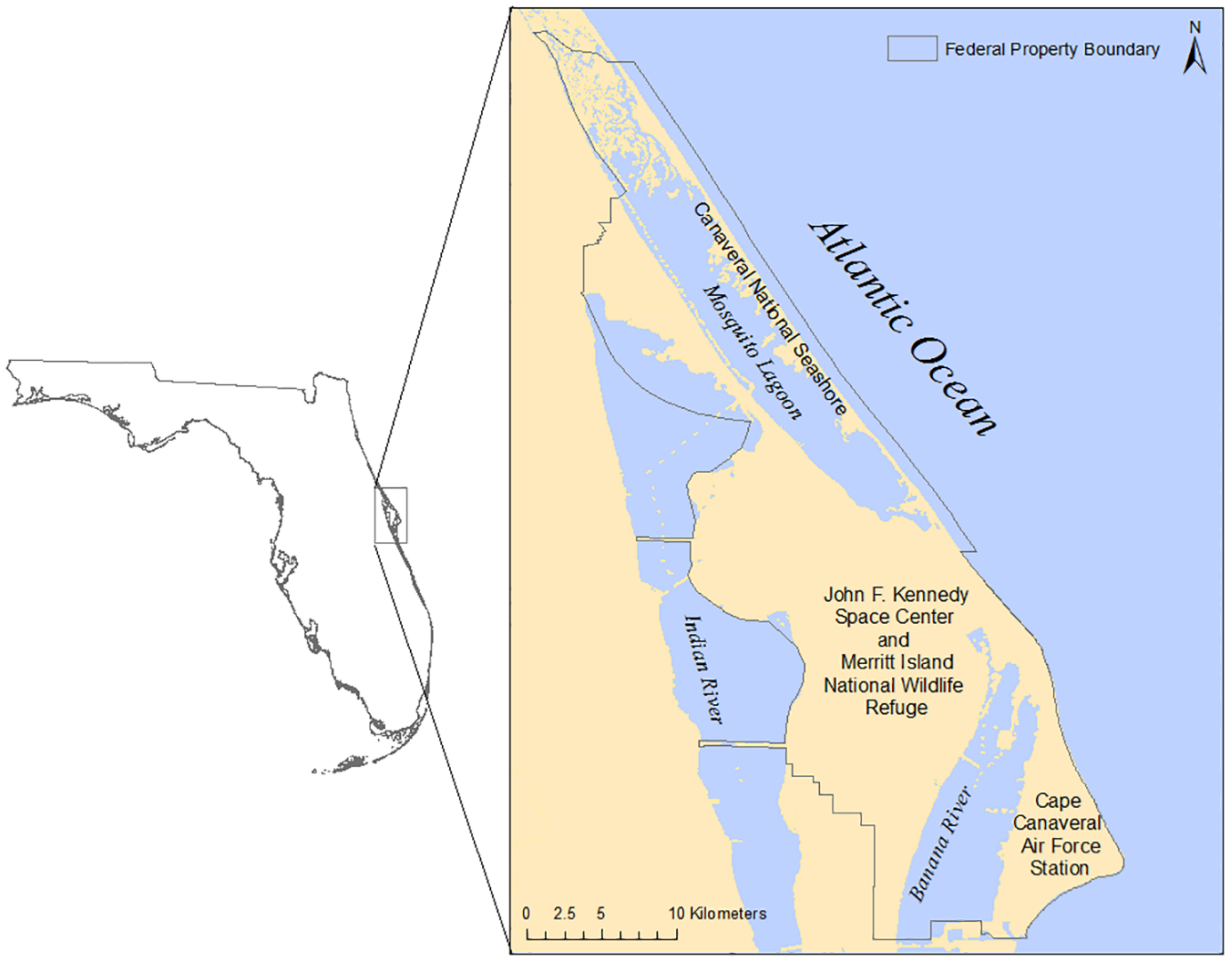
The site map for the study location. The map includes the geographic locations of the federal properties within the Cape Canaveral Barrier Island Complex.

CCBIC is part of the Indian River Lagoon (IRL) ecosystem and includes three estuarine water bodies, Mosquito Lagoon, Banana River Lagoon and Indian River Lagoon (Fig 2). This transitional geographic location between the Caribbean and Carolinian zoogeographic provinces contributes to the area being recognized as one of the most biologically diverse estuarine watersheds in North America with more than 2,200 different species of animals and 2,100 species of plants (St. Johns River Water Management District (SJRWMD), http://www.sjrwmd.com/indianriverlagoon/fastfacts.html). This area also has the highest number of threatened and endangered species on federal property in the contiguous U.S [25, 26]. Four federal facilities; Kennedy Space Center (KSC), Merritt Island National Wildlife Refuge (MINWR), Cape Canaveral Air Force Station (CCAFS), and the Canaveral National Seashore (CNS) occur on CCBIC. CCBIC has high biodiversity, rich ecosystem services, and roughly 11 billion dollars of national assets for access to space [27]. Tourism in the area, associated with KSC, real-estate, and the natural resources of the IRL and its watershed, produced $3.7 billion in benefits to the regional economy [28].

**Fig 3:**
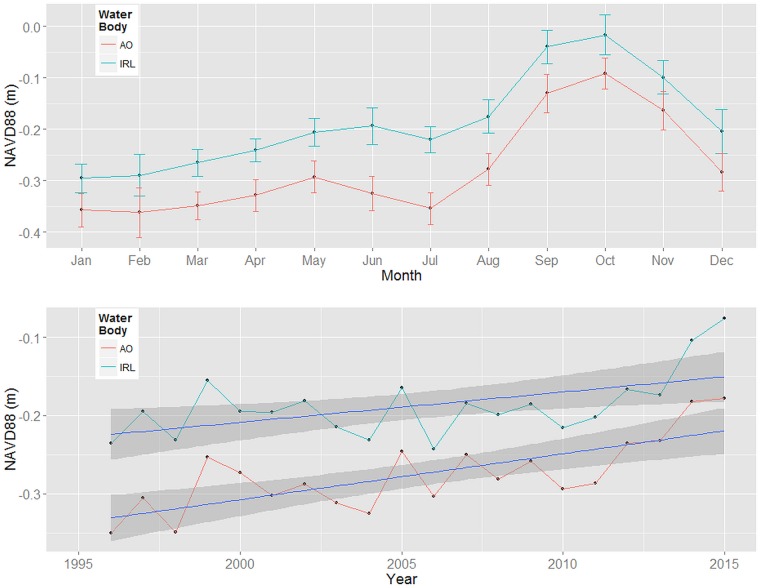
Comparison of monthly and 20 year annual trends in sea level and lagoon level near the Cape Canaveral Barrier Island Complex, FL. Monthly data and annual trend lines include the 95% confidence limits. Data for the Atlantic Ocean (AO) are from the NOAA gauge at Trident Pier (8721604) in Port Canaveral, FL. Indian River Lagoon (IRL) data are from the USGS gauge at Haulover Canal, FL (02248380).

CCBIC was formed as a prograding barrier islands consisting of multiple dune ridges [29]. Land elevations range from sea level to approximately nine meters above sea level. Topography plays an important role in soil formation on CCBIC; soils are mostly sands ranging from well-drained on upland sites to poorly-drained or saline at lower elevations [30]. The climate is humid subtropical with mean annual high temperatures in July (28.0°C) and low in January (15.5°C). Annual precipitation averages approximately 130 cm with 60% of precipitation occurring between June and September (data obtained from the National Atmospheric Deposition Program, FL99). Evapotranspiration rates are high during the spring and summer [31]. The estuary system within CCBIC is micro-tidal due to the physical distance from and small size of the two encompassing inlets: Ponce de Leon Inlet (North) and Sebastian Inlet (South) [32, 33] and accretion rates are low [34]. Sea level and lagoon level fluctuate by approximately 25 cm seasonally (Fig 3), with annual high water during the fall (September/October) and annual low water during late spring (April/May) (Fig 3). Wind driven seiches of similar magnitude may occur throughout the lagoon depending on weather conditions [33]. Since 1996, when instrumentation records began, regional sea level and lagoon level have increased by approximately 11 cm (Fig 3). The rates of increase appear to be accelerating since 2009–2010 following the pattern of other areas of the U.S. east coast [35]. Additional years of monitoring are needed to validate this trend [36]. Differences in annual sea level and lagoon level can be attributed to direct and indirect inputs of rainfall through deposition, runoff and groundwater seepage [37].

### Land cover and elevation analysis

We constructed a stochastic simulation model of proportional changes of vegetation communities due to changes in relative elevation caused by SLR. We defined a vegetation community as an assemblage of dominant plant life forms organized in relation to elevation and salinity. Our assumption was that the relationship between vegetation communities and elevation would persist following SLR of between 0.2 m and 1.2 m as predicted by the NASA Climate Adaptation Science Investigators (CASI) downscaled projections through the later part of the 21^st^ century (Table 1) [38].

**Table 1:** Downscaled sea-level rise projections for the Cape Canaveral Barrier Island Complex provided by the NASA Climate Adaptation Science Investigators (CASI) program [38].

Baseline (2000–2004)	Low Estimate (10^th^ Percentile)	Middle Range (25^th^ to 75^th^ Percentile)	High Estimate (90^th^ Percentile)
2020s	5.1 cm	7.6 to 15.2 cm	20.3 cm
2050s	15.2 cm	22.9 to 43.2 cm	63.5 cm
2080s	25.4 cm	38.1 to 83.8 cm	124.5 cm

Projections are based on a 4-component approach that incorporates both local and global factors. The model-based components are from 24 Global Climate Models and two Representative Concentration Pathways. Shown are the low-estimate (10th percentile), middle range (25th percentile to 75th percentile), and high-estimate (90th percentile). Projections are relative to the 2000–2004 base period.

To obtain elevations for our model input, we derived a Digital Terrain Model (DTM) of the study area reflecting ground elevations (i.e., bald earth) from LiDAR data. LiDAR data for Volusia County acquired during 2006 and Brevard County acquired during 2007 were obtained from the National Oceanic and Atmospheric Administration (NOAA) Digital Coast (https://coast.noaa.gov/digitalcoast/). The DTM was generated from LiDAR points classified as terrain by converting the points directly to raster without interpolation at a cell size of three meters. Because of the sometimes limitated ability of LiDAR to penetrate the plant canopy, terrain extraction may be problematic in vegetated areas. The reported vertical accuracy of LiDAR points obscured by vegetation, as compared to field acquired quality control data, for Brevard [39] and Volusia [40] Counties is < = 0.32 m.

A land cover map of the study area was generated from color infra-red aerial imagery acquired on November 09, 2010 supplemented with imagery from February 2007 and 2009 to identify hardwood wetlands which have a conspicuous leaf-off period during winter. The DTM was used to identify drainage patterns to help separate wetlands and upland communities. Land cover classes consider hydration, salinity, life form and species composition.

Nineteen of these land cover types consisted of natural communities and were selected for inclusion in this study (S1 Text). Using a Geographic Information System (GIS), we obtained the elevation from the DTM for each pixel and extracted the corresponding land cover type. We created density plots of elevation within land cover types to identify groups that exhibited similar elevation trends and combined them into nine vegetation communities used for our modeling (S1 Text). We chose to reduce the number of the community types to keep the model more tractable. We did this by grouping community types that had very similar elevation profiles. These community types also made sense from a management perspective because of the coarse tools available for managing natural areas on CCBIC (prescribed fire and hydrology). However, our method is amenable to including more (or less) community types.

We used generalized least square linear regression on a subsample (n = 10000) of pixels to identify the strength of the relationship between elevation and these nine vegetation communities. Because both the elevation and the community type of spatially close pixels was not independent, we included spatial structure (latitude and longitude) in model residuals to account for any spatial dependence between pixels. We also allowed the variance to differ between communities to better fit patterns in the data [41, 42]. Residuals were modeled as an exponential function of the distance between pixels with parameters estimated from the data. For the community specific variance, each community was allowed to have a different estimated variance.

### Community change model

We based our SLR simulation on an adaptation of the naïve Bayes classifier. The principle behind this classifier is that given a set of objects each with a class character and a vector of measurements, a good way to classify the objects is to base the classification on the probability distributions of measures within the classes [43]. Our class character was community type and the measure vector consisted of only one measure, elevation. To conduct the SLR simulation we first calculated for each pixel on CCBIC the change in relative elevation that would occur due to the chosen level of SLR. The relative elevation change was determined in reference to the mean lagoon high water (MLHW) and all reported elevations from the simulations are in MLHW. Under current conditions the MLHW occurs at approximately 0.0 m NAVD 88 based on the 5 year average for Haulover Canal (Fig 3). With a 0.2 m rise in sea level, the MLHW would occur at 0.2 m NAVD 88. Therefore, a pixel on CCBIC that has an elevation above MLHW of 1.2 m under current conditions would shift to 1.0 m above MLHW under the 0.2 m SLR scenario. Then, for each pixel, we randomly selected a new community type based on the distributions of community occurrence for that elevation in the current landscape. The new landscape represents a realization of the occurrence of vegetation community types resulting from SLR, based on the relationship between elevation and community type in the current landscape.

The input data for our simulation included the two raster data layers previously described; the DTM and the land cover, with aligned 3 m resolution pixels. From these data layers we calculated the current proportions for each of the nine communities at each elevation. Pixels below mean lagoon water (MLW) were assigned to the absorbing state of open water. MLW is approximately 0.2 m lower than the MLHW level occurring at an elevation of -0.20 m NAVD as determined from the long-term average at Haulover Canal (Fig 3) and was used as a lower boundary for assigning a community type to a pixel due to the limitations of the DTM caused by inability to penetrate water. The probability distributions used to assign new community types to pixels following elevation change were developed from the empirical distributions of elevations within the community types as follows. First we stepped through all elevations from the lowest to the highest, calculating the relative proportion of each community type at that elevation. Because the elevation data was sparse in some regions of the distributions of elevations within some community types, we smoothed the distributions of community types by calculating kernel density estimates of the distributions of elevations within each community type. We then calculated the proportions of each community type for each elevation from the kernel density estimates. To avoid anomalies due to discretization we used an arbitrarily small step size determined by trial and error (0.001 m). This procedure resulted in a set of probability distributions of elevations within community types, coded as a matrix (termed the community-elevation proportion matrix) with rows corresponding to the elevation steps, columns corresponding to the community types, and matrix entries consisting of the proportion of each community type for each row (elevation step).

The simulation was conducted by looping through each pixel in the input data and calculating a new relative elevation based on the amount of SLR. A new community type was chosen from a multinomial distribution with outcome class probabilities determined from the community-elevation proportion matrix. During each simulation we kept track of the number of pixels that changed from a community type to each of the other community types, resulting in a 9 x 9 contingency matrix. We also recorded the final proportions of each community type summed over the entire study area. We ran four different levels of elevation change including one with no change and three with relative elevation changes of 0.2 m, 0.4 m, and 1.2 m to simulate SLR scenarios. To better understand the effect of stochasticity on results we ran 1000 replicate simulations for each elevation change, and report the median and end-points of the central 95% of final proportions. We also investigated the effect of sample sizes by randomly selecting subsamples of N = (10^6^, 10^5^, 10^4^, 500, 250) pixels from the original data, and performing 500 replicate simulations for each of the 4 elevation change levels with each sub-sample size. Code to conduct the simulations in R is available upon request from the senior author.

## Results

### Land cover and elevation analysis

The vegetation communities were segregated along the elevation gradient on CCBIC. The estimated mean elevations (m above MLHW) from the regression analysis were well separated for most of the nine vegetation communities (Nagelkerke's R^2^ = 0.93) (Table 2) [44]. There was support for including a spatial residual structure (ΔAIC = 8274 between the models with and without the correlation structure). For the community specific variance, mangrove, wetland scrub—shrub and salt marsh had much lower variance than did the other communities. Cabbage palm and pine flatwoods had slightly lower variances than the rest, which were very similar. The estimates of mean elevation for mangrove and wetland scrub shrub overlapped. Oak scrub was the community that occurred at the highest elevations and saltwater marsh occurred at the lowest elevations (Table 2).

**Table 2:** The estimated mean elevations (95% confidence interval) were well separated for the nine vegetation communities on the Cape Canaveral Barrier Island Complex, FL. The parameter estimate are from a generalized least square model of elevations for each community with spatial correlation structure. Stratum SD is the modeled standard deviation which was allowed to vary by community. The model also allowed the errors to be spatially correlated according to an exponential function with coefficients fit to the data.

Community	Mean elevation estimate (m)	95% Confidence interval	Stratum SD
Saltwater Marsh	0.14	(0.05, 0.23)	0.55
Wet Scrub-Shrub	0.34	(0.2, 0.49)	0.87
Mangrove	0.32	(0.15, 0.48)	1
Cabbage Palm	0.78	(0.55, 1)	1.26
Freshwater Wetland	0.77	(0.58, 0.95)	1.07
Hardwood Hammock	0.88	(0.65, 1.11)	1.40
Pine Flatwoods	1.09	(0.91, 1.28)	1.00
Upland Forest	1.83	(1.46, 2.2)	1.80
Oak Scrub	1.96	(1.67, 2.25)	1.65

**Fig 4:**
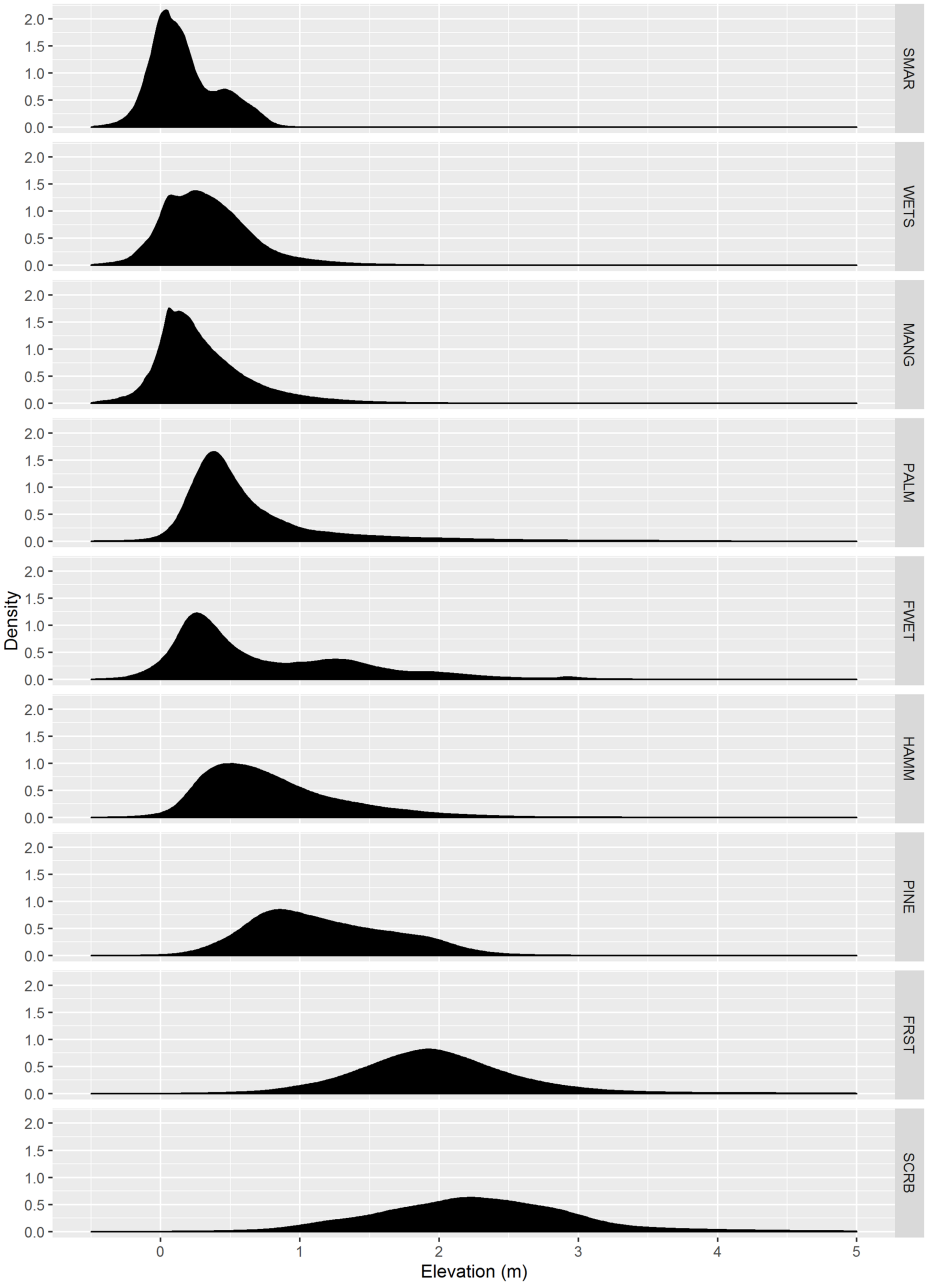
Density plots of elevation for the 9 community types included in models of land cover change due to sea-level rise on the Cape Canaveral Barrier Island Complex. SMAR = Saltwater Marsh, WETS = Wet Scrub-Shrub, MANG = Mangrove, PALM = Cabbage Palm, FWET = Freshwater Wetland, HAMM = Hardwood Hammock, PINE = Pine Flatwoods, FRST = Upland Forest, SCRB = Oak Scrub.

Some of the vegetation communities, e.g., oak scrub and forest, had a broad elevation range while other community types had narrow elevation ranges (i.e., cabbage palm). Freshwater and saltwater marshes had dual elevation peaks (Fig 4).

**Fig 5:**
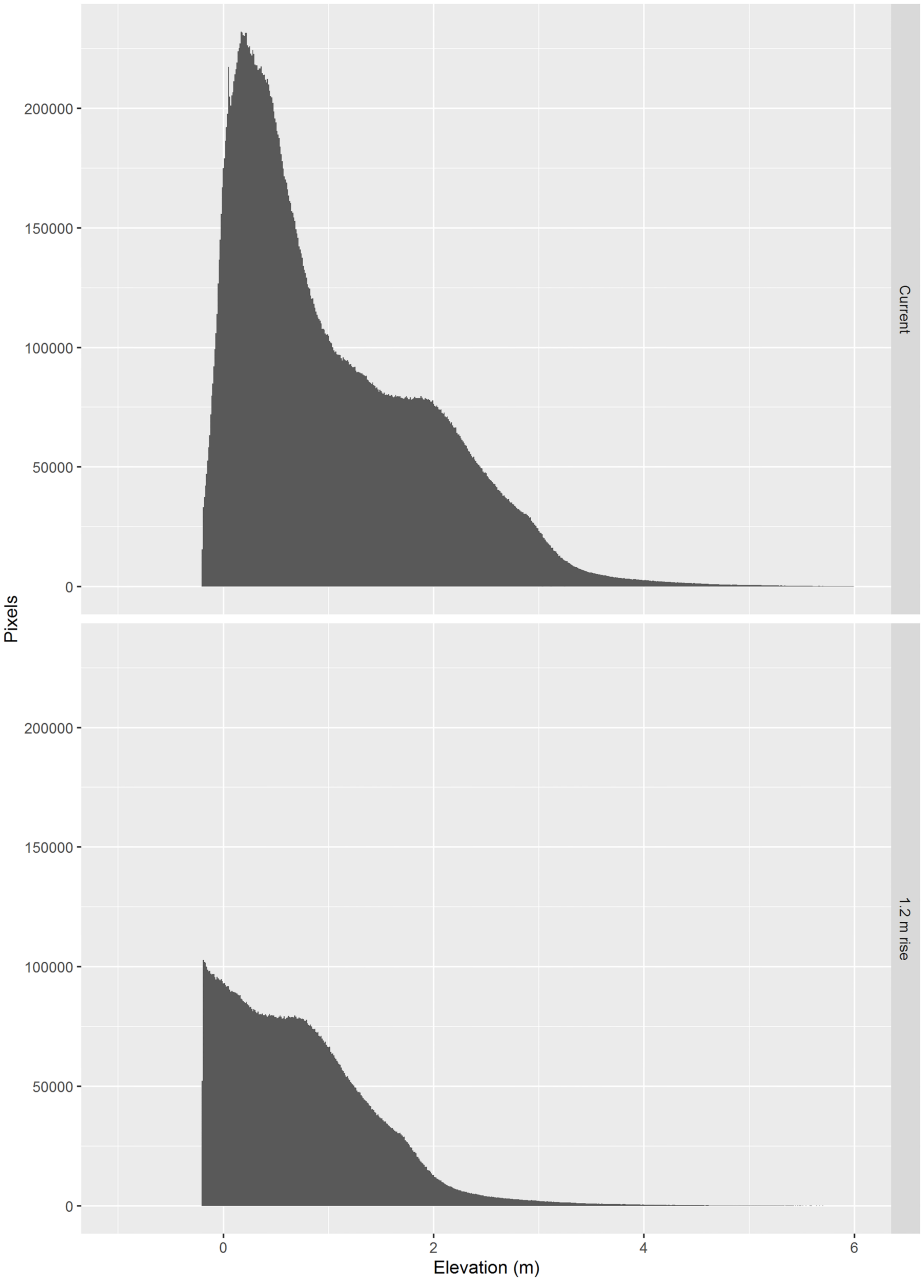
The distribution of landscape units (pixels) by elevation that are above mean lagoon high water elevation on Cape Canaveral Barrier Island Complex for current sea-level (above) and 1.2 m sea-level rise (below). The landscape units that are below -0.2 m in elevation were not included because these are mainly open water lacking emergent vegetation. For the 1.2 m sea-level rise scenario over half of the landscape units have been absorbed and the peak elevation has shifted.

The elevation distribution of landscape units (pixels) above MLHW changed dramatically between SLR scenarios (Fig 5). This was due to the interaction between the topography of the landscape which slopes gradually towards the ocean and estuary, and the absorbing nature of the boundary at the lower range of elevation (open water without emergent vegetation). Under current conditions, the mean land elevation is 1.05 m MLHW. This decreased to 0.74 m MLHW under the 1.2 m SLR scenario as the land area decreased in size (Fig 5). More striking was the change in shape of the elevation profile (Fig 5) as more than half (58%) of the landscape was inundated (transitioned to the absorbing state). The peak density of elevations shifted from 0.19 m MLHW for the current sea-level landscape to -0.13 m MLHW for the landscape with 1.2 m SLR. Much of the area with higher elevations, where upland communities occur, was no longer above water under this SLR scenario.

### Model validation by simulation for baseline with no SLR

**Fig 6:**
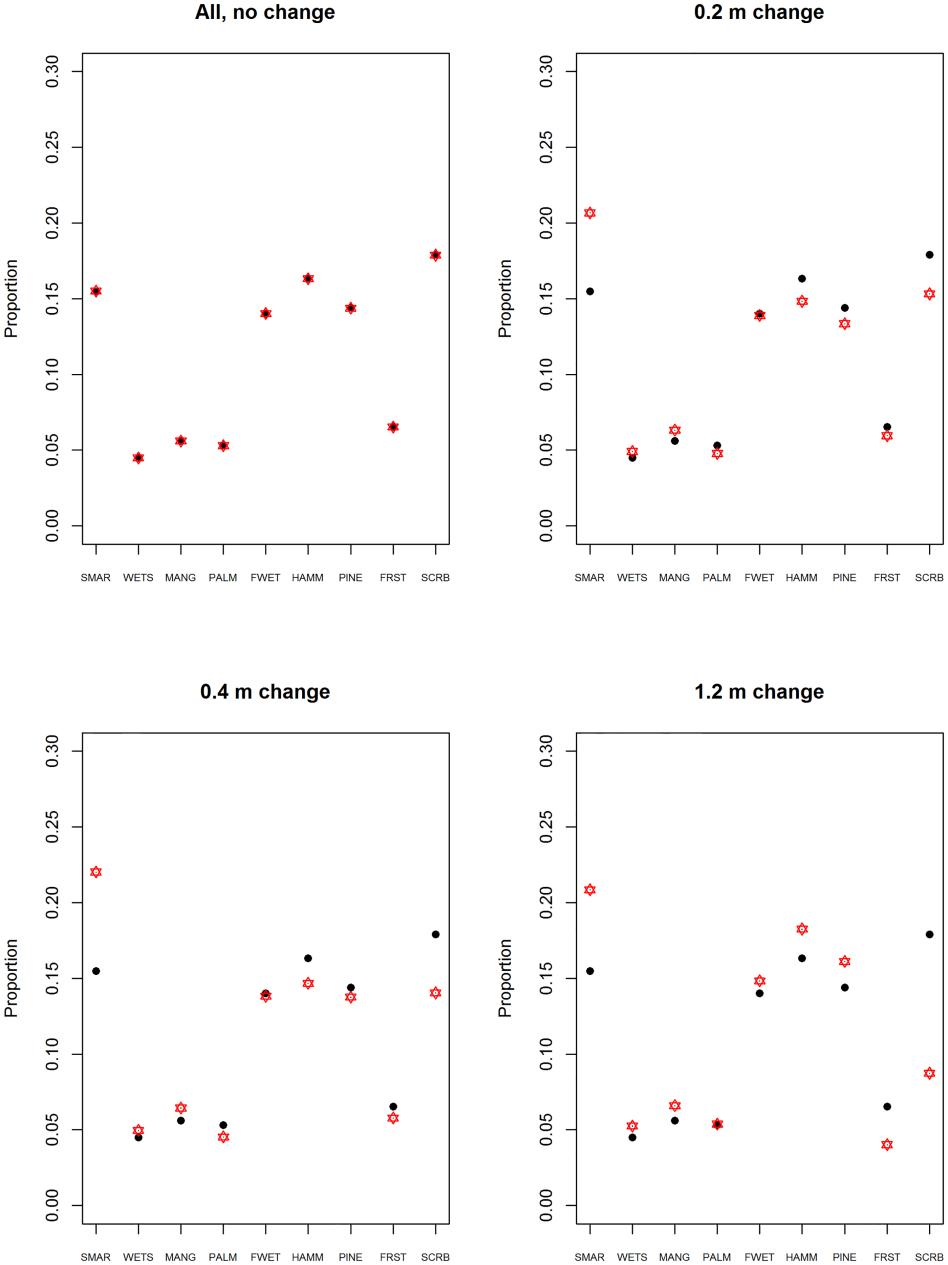
Proportion of each of 9 community types for 4 sea-level rise scenarios on the Cape Canaveral Barrier Island Complex. The black dots are the current proportions of each community type on the CCIBC landscape. The red symbols show the 95% confidence interval for predicted proportion under scenario. The No change scenario is the simulated results of applying the method to the current landscape with no relative elevation change due to sea-level rise; a baseline to judge how the model performs. The proportion that move to the absorbing state are not included in this analysis. SMAR = Saltwater Marsh, WETS = Wet Scrub-Shrub, MANG = Mangrove, PALM = Cabbage Palm, FWET = Freshwater Wetland, HAMM = Hardwood Hammock, PINE = Pine Flatwoods, FRST = Upland Forest, SCRB = Oak Scrub.

When we used current sea-level as input (no relative elevation change due to SLR) our naïve Bayes Monte Carlo modeling procedure predicted vegetative community proportions that were very close to those observed (Fig 6), providing confidence in our methods. Despite the nearly exact agreement of the proportions, the actual amount of pixels that switched community type was quite high (Table 3). This was consistent with how our model works; the community type for each pixel is selected based on the probability of community occurrences for the elevation of the pixel. Thus our model is not spatially explicit but rather models the collective behavior of all of the pixels. For all SLR scenarios, the range of the predicted proportions of community types across replicate simulations was very narrow (Fig 6).

**Table 3:** Contingency table from the simulation run under current sea-level conditions (no change) at Cape Canaveral Barrier Island Complex, FL. The community transitioned from is the current community that exists at each pixel. The community transitioned to is the community that pixel transitioned to during the simulation. Each value in the table is a percent of the total pixels (33,935,656 pixels). Although, the total percentage of pixels for each community was close to the same, there was a high percentage of pixels that shifted community type.

	**Community Transitioned To**									
**Community Transitioned From**	SMAR	WETS	MANG	PALM	FWET	HAMM	PINE	FRST	SCRB	**Percent Original Pixels**
SMAR	6.55	1.32	1.82	0.86	2.46	1.84	0.57	0.02	0.04	**15.48**
WETS	1.33	0.35	0.45	0.34	0.79	0.80	0.35	0.03	0.05	**4.49**
MANG	1.82	0.45	0.59	0.38	0.97	0.88	0.40	0.03	0.07	**5.60**
PALM	0.86	0.34	0.38	0.46	0.93	1.19	0.68	0.13	0.33	**5.30**
FWET	2.46	0.79	0.97	0.93	2.38	2.60	2.00	0.59	1.31	**14.02**
HAMM	1.85	0.81	0.88	1.19	2.60	3.95	3.20	0.60	1.25	**16.31**
PINE	0.57	0.35	0.40	0.68	2.00	3.20	3.82	1.15	2.21	**14.38**
FRST	0.02	0.03	0.04	0.13	0.59	0.60	1.15	1.11	2.88	**6.54**
SCRB	0.04	0.05	0.07	0.33	1.31	1.25	2.22	2.88	9.73	**17.89**
**Percent Transition Pixels**	**15.50**	**4.49**	**5.60**	**5.30**	**14.02**	**16.31**	**14.37**	**6.53**	**17.87**	

### SLR scenarios

**Fig 7:**
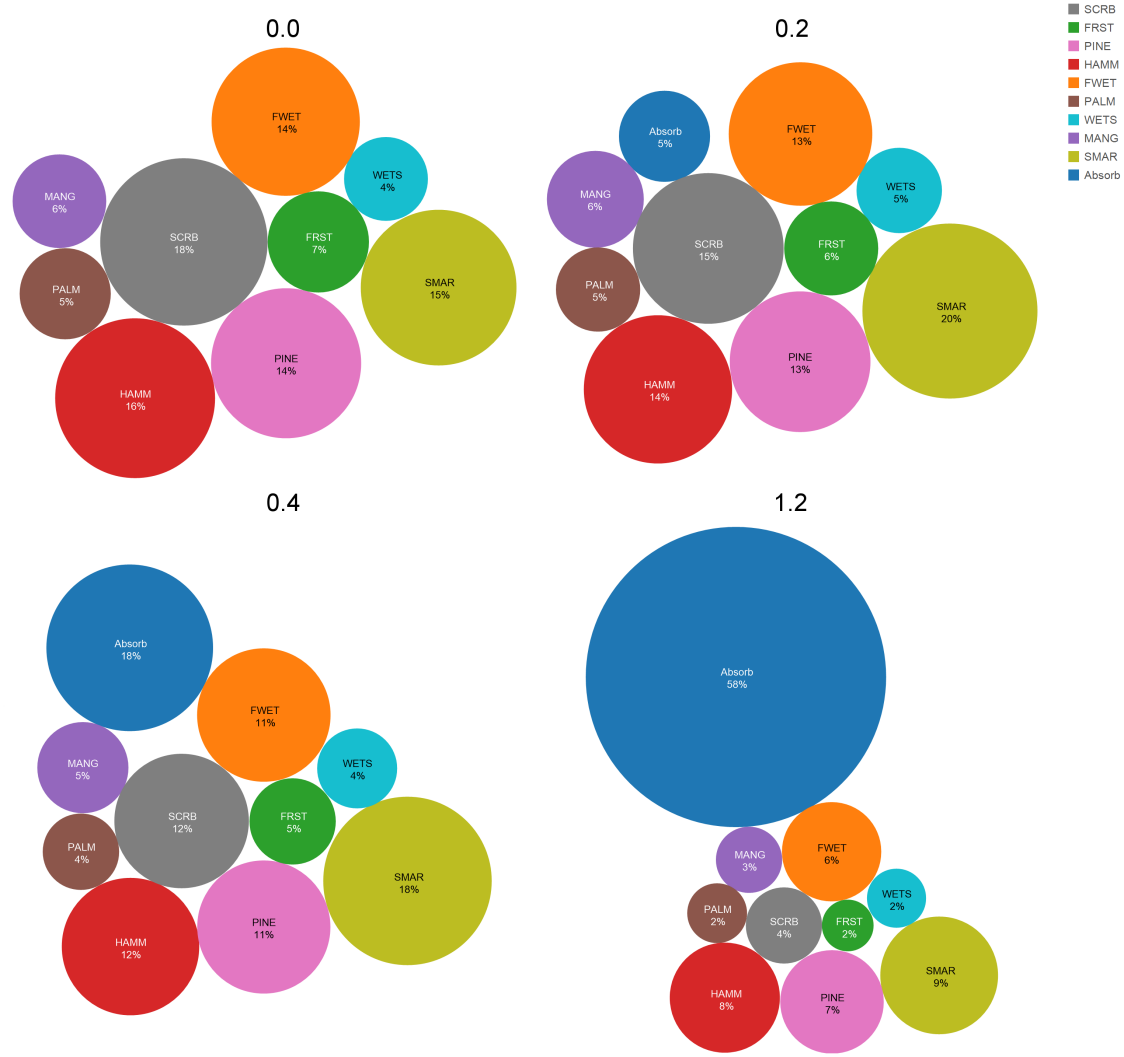
The change in community proportion for the current conditions and the three sea-level rise scenarios on Cape Canaveral Barrier Island Complex. This proportion is determined based on the total number of pixels in the current scenario. SMAR = Saltwater Marsh, WETS = Wet Scrub-Shrub, MANG = Mangrove, PALM = Cabbage Palm, FWET = Freshwater Wetland, HAMM = Hardwood Hammock, PINE = Pine Flatwoods, FRST = Upland Forest, SCRB = Oak Scrub.

The greatest decreases in community area under the varying SLR simulations occurred in the xeric communities with oak scrub decreasing 79% and upland forests decreasing 73% with a 1.2 m SLR (Table 4, Fig 7). The mesic and hydric communities also decreased substantially with a 1.2 m SLR, as much of the lower elevations transitioned to the absorbing state of open water (Table 4, Fig 7). The trends were similar for the 0.2 and 0.4 m sea-level scenario. Saltmarsh increased in area under both the 0.2 m and 0.4 m SLR. An increase in area occurred for wetland scrub-shrub and mangrove under the 0.2 m scenario, but both communities decreased in area when SLR increased beyond that level. Approximately 58% of CCBIC’s landscape included in this study shifted to elevations below -0.2 m MLHW under the 1.2 m SLR scenario; this elevation is below where existing terrestrial vegetation occurs and thus will likely become open water, since accretion rates are unlikely to be enough to keep up with this rate of change [34, 45]. A 0.4 m rise in sea level resulted in just less than 18% of the land area shifting to open water while only 5% shifted to open water with a 0.2 m rise in sea level.

**Table 4:** Percent change (compared to current conditions) for each community type in the total area (pixels) of each land cover type under the different sea- level rise scenarios on Cape Canaveral Barrier Island Complex. Negative numbers represent loss of area (pixels) of a community type. Within a scenario the total percentages do not add to 0 because there was a net loss of pixels to the absorbing state of open water.

SLR scenario	Saltwater Marsh	Wet Scrub Shrub	Mangrove	Cabbage Palm	Freshwater Wetlands	Hardwood Hammock	Pine Flatwoods	Upland Forest	Oak scrub
0.2	26.37	3.80	6.98	-14.56	-6.25	-13.93	-12.10	-13.71	-18.93
0.4	17.03	-9.01	-5.35	-29.66	-18.94	-26.06	-21.28	-27.28	-35.46
1.2	-42.87	-50.29	-49.99	-56.92	-55.09	-52.56	-52.46	-73.94	-79.26

**Fig 8:**
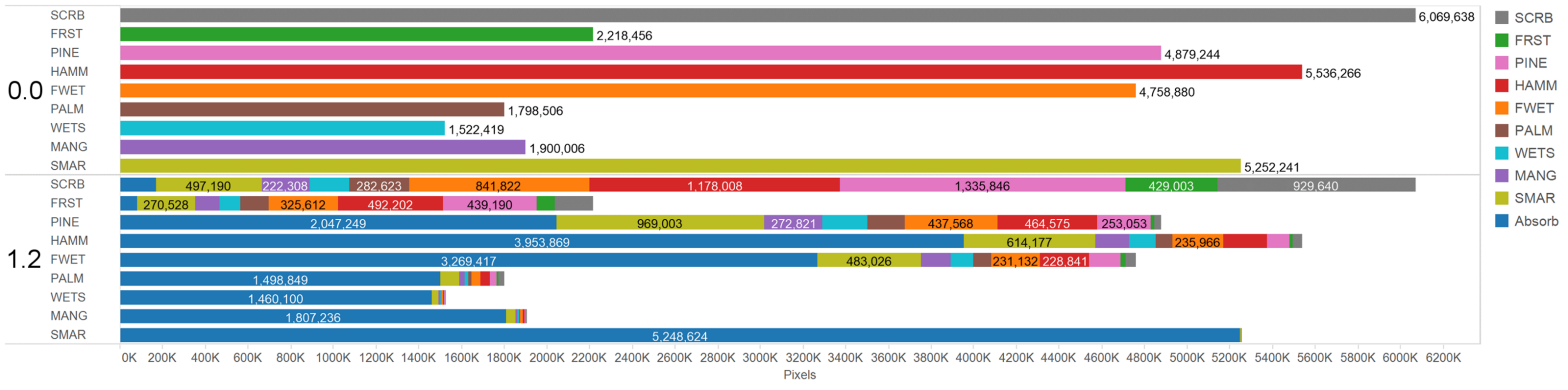
The current number of pixels a community occupies under current conditions (above) and the community transitions that occur under the 1.2 m sea level rise simulation (below). SMAR = Saltwater Marsh, WETS = Wet Scrub-Shrub, MANG = Mangrove, PALM = Cabbage Palm, FWET = Freshwater Wetland, HAMM = Hardwood Hammock, PINE = Pine Flatwoods, FRST = Upland Forest, SCRB = Oak Scrub.

The dominant community transition was conversion to saltwater marsh across all SLR scenarios. In the 0.2 m sea-level scenario 40% of mangrove and 37% of wet scrub-shrub transitioned to saltmarsh (Table 5). A larger percentage of mangrove and wetland scrub-shrub transitioned to the absorbing state in the 0.4 m SLR scenario than to saltmarsh (Table 5). Twenty-eight percent and 37% of cabbage palm transitioned to salt marsh in the 0.2 m and 0.4 m scenarios. The net gain of saltmarsh from mesic and upland communities exceeded the net loss to the absorbing state (Fig 8; Table 5) in both the 0.2 m and 0.4 m scenarios. All of the original salt marsh (100%) was predicted to transition to the absorbing state in the 1.2 m scenario. Under this scenario, 96% of wetland shrub-scrub, 95% of mangrove, 83% of cabbage palm, 71% of hammock and 69% of freshwater wetland transitioned to the absorbing state.

**Table 5:** Contingency tables for the proportional change in community under the varying sea-level rise scenarios. The community transitioned from is the current community that exists at each pixel. The community transitioned to is the community that pixel transitioned to during the simulation. Bold entries give the proportion of each pixels within community type that did not change type.

		**Community Transitioned To**									
**Community Transitioned From**	**SLR Scenario**	Absorbed	SMAR	WETS	MANG	PALM	FWET	HAMM	PINE	FRST	SCRB
SMAR	0.2	0.23	**0.39**	0.07	0.09	0.03	0.11	0.06	0.01	0	0
WETS	0.2	0.1	0.37	**0.08**	0.1	0.05	0.15	0.11	0.04	0	0.01
MANG	0.2	0.11	0.4	0.07	**0.1**	0.04	0.13	0.1	0.04	0	0.01
PALM	0.2	0.01	0.28	0.07	0.1	**0.07**	0.17	0.16	0.08	0.02	0.05
FWET	0.2	0.04	0.26	0.06	0.08	0.05	**0.15**	0.15	0.12	0.03	0.07
HAMM	0.2	0.01	0.19	0.06	0.07	0.07	0.17	**0.21**	0.14	0.02	0.05
PINE	0.2	0	0.08	0.04	0.04	0.06	0.15	0.23	**0.24**	0.06	0.11
FRST	0.2	0	0.01	0.01	0.01	0.02	0.11	0.12	0.21	**0.15**	0.36
SCRB	0.2	0	0	0	0.01	0.02	0.08	0.09	0.16	0.16	**0.47**
SMAR	0.4	0.62	**0.2**	0.03	0.05	0.01	0.06	0.02	0	0	0
WETS	0.4	0.35	0.31	**0.06**	0.08	0.03	0.09	0.06	0.02	0	0
MANG	0.4	0.44	0.27	0.05	**0.06**	0.02	0.08	0.05	0.02	0	0
PALM	0.4	0.09	0.37	0.07	0.09	**0.04**	0.13	0.1	0.05	0.01	0.04
FWET	0.4	0.18	0.26	0.05	0.07	0.04	**0.11**	0.12	0.1	0.02	0.05
HAMM	0.4	0.05	0.27	0.07	0.08	0.06	0.15	**0.16**	0.1	0.02	0.03
PINE	0.4	0.01	0.13	0.05	0.06	0.07	0.16	0.23	**0.2**	0.04	0.07
FRST	0.4	0	0.01	0.01	0.01	0.03	0.12	0.15	0.24	**0.13**	0.29
SCRB	0.4	0	0.01	0.01	0.01	0.02	0.1	0.11	0.19	0.15	**0.4**
SMAR	1.2	1	**0**	0	0	0	0	0	0	0	0
WETS	1.2	0.96	0.02	**0**	0	0	0.01	0	0	0	0
MANG	1.2	0.95	0.02	0	**0.01**	0	0.01	0	0	0	0
PALM	1.2	0.83	0.05	0.01	0.01	**0.01**	0.02	0.02	0.02	0.01	0.01
FWET	1.2	0.69	0.1	0.02	0.03	0.02	**0.05**	0.05	0.03	0.01	0.01
HAMM	1.2	0.71	0.11	0.02	0.03	0.01	0.04	**0.04**	0.02	0	0.01
PINE	1.2	0.42	0.2	0.04	0.06	0.04	0.09	0.1	**0.05**	0	0.01
FRST	1.2	0.04	0.12	0.04	0.05	0.06	0.15	0.22	0.2	**0.04**	0.08
SCRB	1.2	0.03	0.08	0.03	0.04	0.05	0.14	0.19	0.22	0.07	**0.15**

SMAR = Saltwater Marsh, WETS = Wet Scrub-Shrub, MANG = Mangrove, PALM = Cabbage Palm, FWET = Freshwater Wetland, HAMM = Hardwood Hammock, PINE = Pine Flatwoods, FRST = Upland Forest, SCRB = Oak Scrub

The oak scrub community, which occupies the highest driest locations, transitioned primarily to upland forest and pine flatwoods in the 0.2 m and 0.4 m SLR scenarios (Table 5). Over half of the current oak scrub transitioned to mesic and hydric communities (pine flatwoods, hardwood hammocks, and freshwater wetlands) with a 1.2 m rise in sea-level (Table 5, Fig 8). Upland forest transitioned mainly to oak scrub and pine flatwoods with both a 0.2 m and 0.4 m rise in sea-level (Table 5). With a 1.2 m SLR, upland forest transitioned primarily to two mesic communities, pine flatwoods and hardwood hammocks (Table 5, Fig 8). Very little of the upland communities shifted to the absorbing state under the 1.2 m SLR scenario (Table 5).

**Fig 9:**
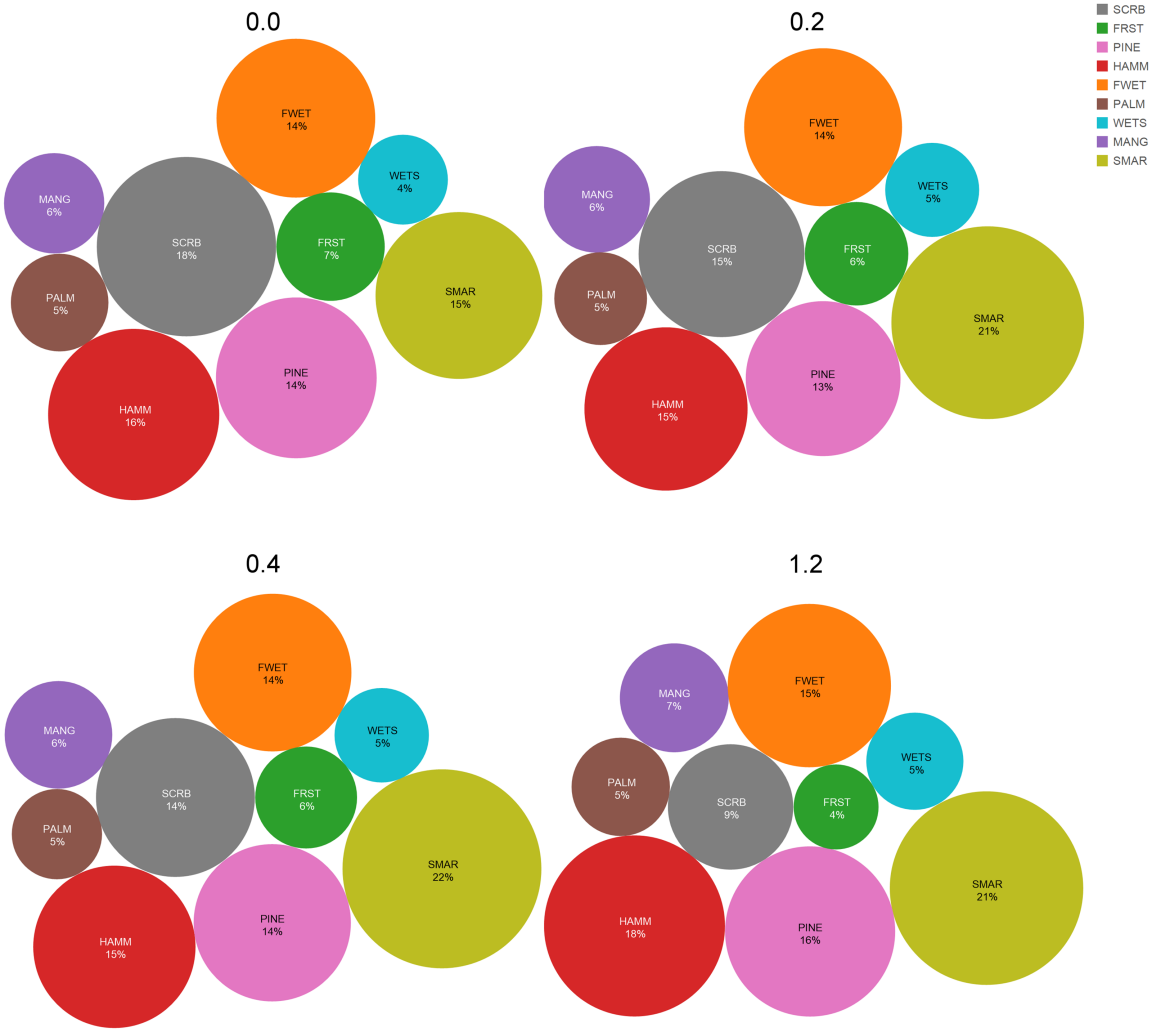
The change in community proportion with the different sea level rise simulations. This proportion is determined based on the number of pixels that remain part of the terrestrial landscape under each simulation. The pixels that move to the absorbing state are not included in this computation.

Under the 1.2 m SLR scenario, CCBIC’s landscape is less than half of its current extent. However, the proportion of most vegetation communities is similar on the remaining landscape (Figs 6 and 9). The exceptions include the most xeric habitat, oak scrub, which occurs on the highest elevations. Only 9% of the landscape remains oak scrub under the highest SLR scenario, compared to the current conditions in which oak scrub occurs on 18% of the landscape. The proportion of salt marsh on the landscape increases from 15% under current conditions to over 20% with all three SLR scenarios.

Decreasing the number of pixels used in the simulation through subsampling increased the range of predictions across replicate simulations in a predictable pattern (S1–S4 Figs). For all three SLR scenarios, samples of 10^5^ and 10^4^ pixels consistently produced simulations from which the changes in community proportions would be detectable. For smaller subsamples (10^3^, 500, 250 pixels), the resulting simulations varied so much that clear patterns were not evident.

## Discussion

Our approach provided a first approximation of how vegetation communities of barrier islands will respond to SLR based on a simulation process that used current relationships between elevation and vegetation communities to simulate potential future conditions. As a proof of concept, our methodology successfully predicted the current vegetation community proportions on the CCBIC landscape.

When applied to three SLR scenarios, our model indicated that the vegetation communities at the highest elevations were the most susceptible to net loss due to SLR (Figs 7 and 8; Table 4). The loss of the xeric upland communities may seem surprising; however, this may be explained because CCBIC is a barrier island with a limited elevation range. Communities at the highest elevations would likely be compressed from the lower elevation bounds as sea level rises and more mesic communities migrate inland, moving up the elevation gradient; eventually leading to a loss of the more xeric upland communities. The modeled loss of xeric upland habitats with SLR was not unique to our study. Dry undeveloped uplands experienced the greatest loss in area when the MAIM model was used to identify the impacts of SLR along the Potomac River [17].

Although, all current salt marsh was predicted to be lost to open water under the 1.2 m SLR, the total proportion of salt marsh on the landscape remained similar to the current proportion due to transitioning of more xeric and mesic communities into salt marsh. A 42% net loss of salt marsh did occur between the current and the 1.2 SLR scenarios even though proportions remained similar. The modeled loss of marsh under large SLR scenarios has been reported from other studies [18]. Other models have also predicted large reductions of salt marsh with SLR in the Indian River Lagoon [46] and tidal marsh in the Potomac River [17].

Our primary assumption in using this technique was that a strong relationship exists between community occurrence and elevation on CCBIC. This is reasonable in Florida where elevation has been found to serve as a good proxy for depth to water table and communities segregate along the depth to water table gradient [22, 23]. This assumption was supported by the partitioning of communities along the elevation gradient observed in our study area (Table 2).

We acknowledge that other climate change factors may impact vegetation response; however, our model focuses on the change in sea level and its influence on lagoon water levels and depth to the water table beneath CCBIC. Climate change projections indicate that precipitation patterns will be altered in many areas. Intermediate projections for CCBIC suggest that annual precipitation will not vary much from the long-term mean (-2 to +8%); although, variations in seasonality may occur [38]. Increased temperature is also expected to impact vegetation migration. Although we expect to see increased cover in our tropical species with warmer temperatures, a strong temperature gradient is not evident on CCBIC that would lead to upward migration of vegetation communities. Coastal barrier island changes driven by sea level rise, changing storm frequency and intensity, and changing wave climates have a high potential to alter regional geomorphology and associated vegetation community species composition and spatial distributions [47–49]. This highly complex topic is beyond the scope of this investigation.

### Potential impacts

Under all SLR scenarios, important terrestrial communities were lost to open water with over half of the land area submerged with a 1.2 m SLR (Figs 7 and 8). This decrease in land area occurred across all vegetation communities under the highest SLR modeled. Such decreases in area would have a significant impact on the existing populations of the plants and wildlife dependent on these vegetation communities at CCBIC. Most of the vegetation communities important for threatened and endangered plants and wildlife on CCIBC are predicted to decrease in occurrence with even a moderate 0.2 or 0.4 m SLR, with the exception of salt marsh which expands under these scenarios due to the conversion of other hydric and mesic vegetation communities. Marsh expansion under low to intermediate SLR has been modeled in other coastal communities [17]. Our modeled marsh area under 0.2 and 0.4 m SLR may be underestimated since accretion rates were not included in our model; therefore, we may overestimate the amount of salt marsh that transitioned to open water under these scenarios. The vulnerability of salt marshes to SLR may be overestimated when geomorphic and biophysical processes are not included in models [18, 50]. Increased accretion rates have been found to occur with intermediate SLR; however, this is dependent on the amounts of suspended sediments available and the tidal range [18]. The micro-tidal nature of the IRL, the low accretion rates, and the impact of impounded wetlands on CCBIC may hinder the ability of salt marshes on CCBIC to withstand intermediate SLR [45].

Our results have important implications for many threatened and endangered species on CCBIC. Forty plant species that occur on CCBIC are listed as threatened or endangered [25]; these species occur primarily in hammocks, freshwater marshes, coastal dunes and oak scrub which exhibited decreases under all SLR scenarios. One hundred nine species of wildlife have been identified as endangered or potentially endangered on CCBIC [25], and the area provides important habitat for the U.S. population of 12 species and for Florida populations of an additional 17 species [25]. Two species, Florida Scrub-jay and the Southeastern Beach Mouse, have populations of global importance on CCBIC [25]. Significantly, two (oak scrub and pine flatwoods) of the three vegetation communities required by the highest number of vulnerable species for which CCBIC is important were predicted to decrease in area under all three of the SLR scenarios. The third community, salt marsh, increased in area under the 0.2 m and 0.4 m but decreased in area under the 1.2 m SLR scenario. These findings suggest that even small changes in sea level may have potentially large impacts on vulnerable species on CCBIC.

There are several implications of SLR on CCBIC that we did not cover in this study. CCBIC contains billions of dollars of national infrastructure. By focusing on the natural areas and the ecosystem services provided by those areas we have neglected to include the cost, both in lost infrastructure and national assets, in our model. Previous modeling exercises have focused on the impact of SLR on these assets while not addressing the impacts on the natural areas [38].

### Alternate drivers and zonation

In some cases elevation may not be the main variable that determines vegetation communities. For example, mangroves and saltwater wetland scrub-shrub overlapped in both their mean elevations and their elevation ranges. These communities may have been shaped more by the frequency of fire and freezes than by elevation, because frequent fire prevents the encroachment of mangroves and other trees by limiting seedling establishment [51] and mangroves are sensitive to hard freezes [52]. We did not include fire or freezes in our models but we acknowledge the role that both play in determining vegetation communities. If saltmarsh and mangrove communities successfully move into areas we predicted to become open water, the implications for the upland communities would not change. However, our results may overestimate salt marsh and underestimate mangroves since freezes are not considered.

The occurrence frequencies of both freshwater wetland and salt marsh across the elevation range exhibited dual peaks. The dual peaks in the freshwater wetlands may have been partially caused by perched marshes that occur in the ridge swale matrix of CCBIC. The swales in this matrix are at a higher elevation than the lower-lying freshwater marshes. The dual peaks might instead have been caused by the lack of resolution within the community types (i.e., we grouped two separate communities into one category). We know that there is strong zonation within saltmarsh communities that is dictated by small changes in elevation [53]. The dual peaks in the saltmarsh might be reflecting the zonation of different saltmarsh communities along the elevation gradient.

The east central Florida landscape has low topographic relief; centimeter elevation changes can separate vegetation zones. Our simulation of current conditions provided an accurate representation of each community’s total number of pixels on the current landscape; however, there was a lot of transition between communities. This is because our technique is based on the probability of finding each community type in these subtle elevation zones where there is overlap between communities. Our model is based solely on the probability of each community occurring within a specific elevation zone; there is no spatial aspect to our model. In reality, pixels near each other are more likely to belong to the same community type [54]. For instance, if a pixel contains oak scrub there is a high probability that the pixels surrounding it are also oak scrub. Including spatial information would allow us to examine how the distribution of these communities on the landscape may change with SLR; however, it also adds complexity to the model. Further modeling is needed to understand the specific locations of vegetation communities that may persist under SLR and which need additional protection and management.

### Implications for future work

We believe that our procedure can provide a rapid first approximation of the changes in the distribution of community types for other low lying coastal regions where there is a strong relationship between elevation and community type, provided that quality land cover data and digital elevation models are available. However, as seen with our sample size analysis, a large sample size exceeding 10,000 points is required considering the sensitivity of the output to sample size. We conducted the simulations using very spatially extensive data (N = 33,935,656) which were available, however in many situations it might be possible to sample only a portion of the landscape. Based on our simulations sample sizes on the order of 10,000 pixels would provide enough precision to predict the correct direction of changes to expect. This quantity of data may soon be readily available due to rapidly increasing availability of unmanned aerial vehicle systems. The precision decreased as the SLR increased, meaning that the uncertainty is greater for the more extreme effects of climate change, making mitigation planning more difficult. Many factors, in addition to SLR and changing hydrology, will impact coastal and barrier islands, often in complex, dynamic and non-linear changes to the ecological envelop of current plant communities. These factors include sediment/soil accretion and erosion rates, changes in soil organic matter and associated geochemistry, depth to water table, salt water intrusion, drought, flooding, and microbial community shifts [48, 55]. These types of changes are not easily or reliably modeled by simple non-spatial approaches, especially when occurring simultaneously. In the next phase of this research we plan to incorporate model output from the FEMWATER model [56] (salt water intrusion, response to changing rainfall volume etc.) with current knowledge regarding the spatial distributions of existing vegetation communities. This may be accomplished utilizing spatially explicit agent based models (or other kinds) to better forecast potential vegetation community responses to a changing climate.

## Supporting information

S1 Text Land cover type descriptions and community groupings. Land cover types highlighted in black were not combined with any other land cover types in our community analysis. Land cover types that were grouped into communities are highlighted by the same color. Blue = Freshwater wetlands, Orange = Oak scrub, Green = Pine Flatwoods, Red = Upland Forest.
(DOCX)

S1 Fig Simulation results of the effect of number of sample pixels on predictions of the proportion of each community type for the 0.2 m increase in sea-level rise scenarios on the Cape Canaveral Barrier Island Complex. The black dots are the current proportions of each community type on the CCIBC landscape. The red symbols show the 95% confidence interval for predicted proportion under scenario. The proportion that move to the absorbing state are not included in this analysis. SMAR = Saltwater Marsh, WETS = Wet Scrub-Shrub, MANG = Mangrove, PALM = Cabbage Palm, FWET = Freshwater Wetland, HAMM = Hardwood Hammock, PINE = Pine Flatwoods, FRST = Upland Forest, SCRB = Oak Scrub.
(7Z) Click here for additional data file.

S2 Fig Simulation results of the effect of number of sample pixels on predictions of the proportion of each community type for the 0.4 m increase in sea-level rise scenarios on the Cape Canaveral Barrier Island Complex. The black dots are the current proportions of each community type on the CCIBC landscape. The red symbols show the 95% confidence interval for predicted proportion under scenario. The proportion that move to the absorbing state are not included in this analysis. SMAR = Saltwater Marsh, WETS = Wet Scrub-Shrub, MANG = Mangrove, PALM = Cabbage Palm, FWET = Freshwater Wetland, HAMM = Hardwood Hammock, PINE = Pine Flatwoods, FRST = Upland Forest, SCRB = Oak Scrub.
(7Z) Click here for additional data file.

S3 Fig Simulation results of the effect of number of sample pixels on predictions of the proportion of each community type for the 1.2 m increase in sea-level rise scenarios on the Cape Canaveral Barrier Island Complex. The black dots are the current proportions of each community type on the CCIBC landscape. The red symbols show the 95% confidence interval for predicted proportion under scenario. The proportion that move to the absorbing state are not included in this analysis. SMAR = Saltwater Marsh, WETS = Wet Scrub-Shrub, MANG = Mangrove, PALM = Cabbage Palm, FWET = Freshwater Wetland, HAMM = Hardwood Hammock, PINE = Pine Flatwoods, FRST = Upland Forest, SCRB = Oak Scrub.
(7Z) Click here for additional data file.

S4 Fig Simulation results of the effect of number of sample pixels on predictions of the current proportion of each community type on the Cape Canaveral Barrier Island Complex. The black dots are the current proportions of each community type on the CCIBC landscape. The red symbols show the 95% confidence interval for predicted proportion under the no change scenario. The proportion that move to the absorbing state are not included in this analysis. SMAR = Saltwater Marsh, WETS = Wet Scrub-Shrub, MANG = Mangrove, PALM = Cabbage Palm, FWET = Freshwater Wetland, HAMM = Hardwood Hammock, PINE = Pine Flatwoods, FRST = Upland Forest, SCRB = Oak Scrub.
(7Z) Click here for additional data file.
