# Supplementary material for: Modeling vegetation community responses to sea-level rise on Barrier Island systems: A case study on the Cape Canaveral Barrier Island complex, Florida, USA
Source: PLoS One. 2017 Aug 10;12(8):e0182605. doi: 10.1371/journal.pone.0182605 (PMC5552030; doi:10.1371/journal.pone.0182605)
Supplement: S1 Text — Land cover types highlighted in black were not combined with any other land cover types in our community analysis. Land cover types that were grouped into communities are highlighted by the same color. Blue = Freshwater wetlands, Orange = Oak scrub, Green = Pine Flatwoods, Red = Upland Forest. (DOCX) [file pone.0182605.s001.docx]

**S1 Text. Land cover type descriptions and community groupings.** Land cover types highlighted in black were not combined with any other land cover types in our community analysis. Land cover types that were grouped into communities are highlighted by the same color. Blue = Freshwater wetlands, Orange = Oak scrub, Green = Pine Flatwoods, Red = Upland Forest

Marsh - saltwater = Herbaceous wetlands that includes impounded and non-impounded systems. Species composition includes sand cord grass, black rush, salt-tolerant grasses (including salt grass, seashore paspalum, and seashore drop seed), and other species.

Marsh - freshwater = Herbaceous wetlands that include beard grass, sand cord grass, saw grass, cattail, and other species.

Mangrove = Includes white mangrove, black mangrove, red mangrove, and buttonwood. Woody vegetation along levees (classified as ruderal - woody) may contain mangroves along the inundated edge mixed with Brazilian pepper.

Wetland scrub - shrub - saltwater = Vegetation composition consists of low height, generally less than 5m, woody species including sea oxeye, saltwort, glasswort, and other species.

Wetland scrub - shrub - freshwater = Vegetation composition consists of low height, generally less than 5m, woody species including Carolina willow intermixed with other species.

Wetland coniferous / hardwood forest = Mix of conifers, primarily slash pines, and assorted hardwood trees including laurel oak, Virginia live oak, cabbage palm, red maple, American elm, and bay; generally greater than 5m tall, with interlocking canopy.

Wetland hardwood forest = Hardwood trees including red maple, American elm, laurel oak, live oak, cabbage palm, and bay, generally greater than 5m tall, with interlocking canopy.

Oak scrub = Includes scrub oak species (i.e.: sand live oak, myrtle oak, Chapman oak), with scattered saw palmetto, wax myrtle, gallberry, lyonias, other shrub and brush species, intermixed with various types of herbs and grasses. Generally less than 5m tall, with interlocking canopy but may also contain small areas with little or no vegetation.

Scrubby flatwoods = oak scrub with scattered pine trees, primarily slash pine.

Palmetto scrub = Includes saw palmetto, wax myrtle, gallberry, lyonias, other shrub and brush species, intermixed with various types of herbs and grasses. Generally less than 5m tall, with interlocking canopy but may also contain small areas with little or no vegetation.

Pine flatwoods = Palmetto scrub with scattered pine trees, primarily slash pine.

Upland coniferous / hardwood forest = Contains tall oaks and pine trees generally greater than 5m tall with interlocking canopy. Composition may include redbay, laurel cherry, southern red cedar, and cabbage palm.

Upland hardwood forest = Contains tall oaks generally greater than 5m with interlocking canopy and an understory that includes saw palmetto. Composition may include redbay, slash pines, laurel cherry, and cabbage palm.

Cabbage palm = A forest community predominantly cabbage palm and is commonly found as hammock communities on shallow rises within wetland communities generally greater than 5m with interlocking canopy.

Hardwood hammock = A forest community commonly found on shallow rises within wetland communities. Greater than 5m with interlocking canopy and predominantly composed of Virginia live oak with laurel oak, cabbage palm, and American elm.
